# Prevalence of allergen sensitization among patients with allergic diseases in Guangzhou, Southern China: a four-year observational study

**DOI:** 10.1186/2049-6958-9-2

**Published:** 2014-01-15

**Authors:** Bao-qing Sun, Pei-yan Zheng, Xiao-wen Zhang, Hui-min Huang, De-hui Chen, Guang-qiao Zeng

**Affiliations:** 1 State Key Laboratory of Respiratory Disease, National Clinical Research Center for Respiratoy Diseases, Guangzhou Institute of Respiratoy Diseases First Affiliated Hospital, Guangzhou Medical University, 151 Yanjiang Rd, Guangzhou 510120, China; 2 Department of Otorhinolaryngology-Head & Neck Surgery, First Affiliated Hospital, Guangzhou Medical University, Guangzhou 510120, China; 3 Department of Pediatrics, First Affiliated Hospital, Guangzhou Medical University, Guangzhou 510120, China

**Keywords:** Allergy, Distribution, Prevalence, Specific immunoglobulin E

## Abstract

**Background:**

The distribution of allergens may vary with different geographic areas, suggesting the importance of local epidemiological data to support evidence-based prevention and management of allergic diseases. We investigated the distribution of common allergens in allergic patients in Guangzhou, southern China.

**Methods:**

7,047 patients with allergic symptoms were examined for serum sIgE to 15 common allergens in this region, based on the protocol of reversed enzyme allergosorbent test.

**Results:**

4,869 (69.09%) of the subjects tested positive for sIgE to at least one of the 15 common allergens. There was no statistical difference in the overall rate of positive sIgE detection between males (3128/4523, 69.16%) and females (1741/2524, 68.98%). *Der pteronyssinus* and *Der farinae* were the most common aeroallergens, while eggs and cow’s milk the most common food allergens, responsible for higher positive rates of sIgE responses. A good correlation in positive sIgE response was found between *Der pteronyssinus* and *Der farinae*. By age-group analysis, we noted several peaks of sensitization to certain allergens: *Der pteronyssinus*, *Der farinae*, and *Blomiatropicalis* at age between 9 and 12; *Blattellagermanica* and mosquito at age between 15 and 18, cow’s milk before age 3; eggs and flour at age between 3 and 6; crabs and shrimps at age between 12 and 15. Along with older age, there was an ascending tendency in the overall positive rate of sIgE response to house dust mites among subjects who tested positive for sIgE to eggs or cow’s milk.

**Conclusions:**

*Der pteronyssinus, Der farinae*, cow’s milk, and eggs are major allergens in Guangzhou. Sensitization to eggs and cow’s milk is more common at younger age, and then gives place to the increasing prevalence of sensitization to *Der pteronyssinus* and *Der farinae* at older age. Such a sequence of events may be a result of allergy march. Knowledge on the prevalence of allergen sensitization in different age groups would help early diagnosis and intervention of allergic diseases in this large geographical region.

## Background

Over the recent decades the growing epidemic of allergic diseases worldwide has become a health concern, for which the World Health Organization puts high priorities on prevention and treatment. Although mechanisms underlying allergies can be considerably complicated, and still have to be fully understood, the presence of three aspects is recognized in the development of allergic diseases, namely: the allergen, the sensitized host, and the close contact between both. However, distribution of allergens may vary with different geographic areas, local climates, environments and lifestyles. In addition, the prevalence of sensitization to allergens may differ across age groups. This fact suggests the paramount importance of local epidemiological data on allergens to support evidence-based prevention and management of allergic diseases, especially in a country with vast-stretching territory. In this article we present a 4-year observational study on 15 common allergens in the sera of patients with allergic diseases performed in Guangzhou, southern China.

## Methods

### Study population

There were 7,047 consecutive patients referred by pediatricians, pulmonologists, dermatologists and otorhinologists in about 50 Guangzhou hospitals for determination of specific IgE (sIgE) to common allergens in our laboratory, one of major centers responsible for confirmatory diagnosis of allergic diseases by using *in vitro* tests in Guangzhou, the largest city of southern China. These patients were clinically evaluated by their attending physicians to have suspected symptoms of allergies including skin rashes, hives, red or itchy eyes, eczema, stuffy or runny nose, sneezing, and gastrointestinal discomfort after consumption of certain foods. The subjects comprised 4,523 males (64.18%) and 2,524 females (35.82%), with a mean age of 11.95 ± 16.57 years (range: one month to 86 years). All the patients underwent sIgE tests for a local combination of allergens which were most commonly reported in previous local studies [1,2]. The tested allergens included 15 common allergens: 7 aeroallergens [*Der pteronyssinus* (D1), *Der farinae* (D2), *Blomiatropicalis* (D5), dog hair (E5), cat dander (E1), *Blattellagermanica* (I6), and mosquito (I71)] and 8 food allergens [egg (F252), cow’s milk (F2), wheat flour (F4), codfish (F3), peanut (F13), soybean (F14), crab (F23), and shrimp (F24)]. For each patient, 5 ml venous blood was collected by routine phlebotomy and centrifuged at 3000 r/min for 10 min. The supernatants were decanted, labelled and stored in the refrigerator at −20 degrees Celsius until their use in the panel testing of allergens within the next 7 days.

The study protocol was approved by the Ethics Committee, First Affiliated Hospital of Guangzhou Medical University (Approval Number: GYFYY-2007-12-06). Written informed consent was obtained from each adult subject and from the parents or guardians of pediatric patients participating in the present study.

### Laboratory setting of sIgE measurement

Serum sIgE against the pre-designated panel of allergens was determined based on the protocol of reversed enzyme allergosorbent test (REAST) in a fully automated micro-plate and slide processor (Ap22 Speedy, DAS, Rome, Italy) by using the ALLERG-O-LIQ System (Dr. FookeLaboratorien GmbH, Neuss, Germany). The REAST represents a modern approach of immunoassay for detection of sIgE, relying on immobilized anti-IgE in combination with liquid biotinylated allergens. The micro-plate processor (AP22, DAS, Italy) used for REAST has been demonstrated to produce low variations between different instruments and between manipulators. Further advantages also included a short hands-on time, as well as a high through-put rate. On each micro-plate, calibrators with defined concentrations of IgE standardized according to the WHO reference preparation for IgE (WHO 75/702) were simultaneously measured with the test samples to generate a calibration curve.

### Evaluation of sIgE test

The concentrations of sIgE in the test samples were quantified by calculation from the calibration curve in international units per milliliter (IU/mL), and also classified quantitatively as classes 0 through 6 according to the manufacturer’s instructions (Dr. Fooke Laboratorien GmbH, Neuss, Germany). A level of sIgE < 0.35 IU/mL was rated as class 0, ≥ 0.35 to < 0.7 IU/mL as class 1, ≥ 0.7 to < 3.5 IU/mL as class 2, ≥ 3.5 to < 17.5 IU/mL as class 3, ≥ 17.5 to < 50 IU/mL as class 4, ≥ 50 to < 100 IU/mL as class 5, and ≥ 100 IU/mL as class 6. Test with a level of sIgE ≥ 0.35 IU/mL (class 1 or above) was defined as positive.

### Statistical analysis

Data were analyzed using the Statistical Package for the Social Sciences Ver 13.0 (SPSS Inc., Chicago, IL, USA) for Windows. Chi-squared test was used to determine the between-group differences of numerical data. P below 0.05 was considered significant. Kappa test was used to evaluate the agreement in positive sIgE test between allergens. A Cohen’s kappa coefficient, kappa ≥ 0.75 was interpreted as good agreement, and kappa < 0.40 as poor or no agreement. Pearson correlation analysis was used to test the correlation among variables where appropriate.

## Results

### Overall sIgE reactivities to aeroallergens and food allergens in the study population

Out of the 7,047 patients, 4,869 (69.09%) tested positive for sIgE to at least one of the 15 common allergens.

Among the 7 test aeroallergens, *Der pteronyssinus* (D1) and *Der farinae* (D2) were the two major ones responsible for positive sIgE tests. By classification of the sIgE reactivities, most aeroallergens (>85%) were associated with low-class allergic responses (classes 1–2, corresponding to a sIgE level between 0.35 and 3.5 IU/mL), except for D1 and D2 (Table 1). The three cases of ≥ class 5 sIgE response to *Blomiatropicalis* (D5) identified in our study were concomitantly with ≥ class 3 immune responses to *Der pteronyssinus* and *Der farinae*. Among the 109 patients with class 3 or 4 sIgE response to *Blomiatropicalis*, only one was not concomitantly allergic to any other aeroallergen. Dog hair and cat dander were responsible for one case each of class 5 or 6 sIgE response (Table 1). *Blattella germanica* or mosquito was not related to any case of ≥ class 5 sIgE response.

**Table 1:** SIgE reactivities to the 15 common allergens in 7,047 patients

**Allergens**	**Positive cases (%)**	**Classification of sIgE response [n (%)]**		
		**Classes 1–2**	**Classes 3–4**	**Classes 5–6**
*Der pteronyssinus* (D1)	2629 (37.31)	975 (37.09)	1153 (43.86)	501 (19.06)
*Der farinae* (D2)	2559 (36.31)	858 (33.53)	1298 (50.72)	403 (15.75)
*Blomia tropicalis* (D5)	797 (11.31)	685 (85.95)	109 (13.68)	3 (0.38)
*Blattella germanica* (I6)	502(7.12)	474 (94.42)	28 (5.58)	0 (0.00)
Dog hair (E5)	300(4.26)	293 (97.67)	6 (2.00)	1 (0.33)
Cat dander (E1)	297 (4.21)	264 (88.89)	32 (10.77)	1 (0.34)
Mosquito (I71)	282 (4.00)	256 (90.78)	26 (9.22)	0 (0.00)
Cow’s milk (F2)	2926 (41.52)	2047 (69.96)	810 (27.68)	69 (2.36)
Egg (F252)	1587 (22.52)	1341 (84.50)	243 (15.31)	3 (0.19)
Wheat flour (F4)	373 (5.29)	352 (94.37)	21 (5.63)	0 (0.00)
Peanut (F13)	363 (5.15)	331 (91.18)	31 (8.54)	1 (0.28)
Crab (F23)	161(2.28)	133 (82.61)	21 (13.04)	7 (4.35)
Shrimp (F24)	160 (2.27)	127 (79.38)	26 (16.25)	7 (4.38)
Soybean (F14)	139 (1.97)	132 (94.96)	7 (5.04)	0 (0.00)
Codfish (F3)	57 (0.81)	53 (92.98)	4 (7.02)	0 (0.00)

Among the 8 food allergens, cow’s milk and egg were the two major ones leading to allergy. By classification of the sIgE reactivities, all tested food allergens were mostly (>70%) associated with class 1 or 2 allergic response in the patients, with the high-class (≥ 5) response being frequently caused by cow’s milk. The three cases of high-class response to eggs in this study were concomitantly sensitized to cow’s milk (class 3 or above). Other food allergens related to occurrence of high-class allergic responses included peanuts (n = 1), crabs (n = 7) and shrimps (n = 7), but none of wheat flour, codfish and soybean (Table 1).

### Gender-specific sIgE reactivities to aeroallergens and food allergens in the study population

**Figure 1:**
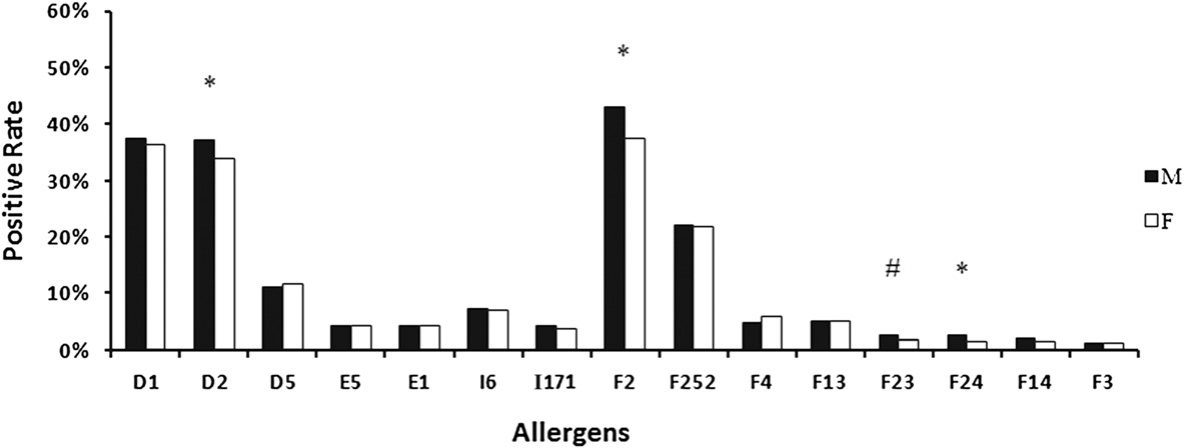
**Positive rate of sIgE antibodies to 15 allergens in both genders.** D1, *Der pteronyssinus*; D2, *Der farinae*; D5, *Blomiatropicalis*; E5, Dog hair; E1, Cat dander; I6, *Blattella germanica*; I71, Mosquito; F252, Egg; F2, Cow’s milk; F4, Wheat flour; F3, Codfish; F13, Peanut; F14, Soybean; F23, Crab; F24, Shrimp. *p < 0.01, ^#^p < 0.05.

Overall, there was no statistical difference in the rate of positive sIgE detection between males (3128/4523, 69.16%) and females (1741/2524, 68.98%) (χ2 = 0.0245, p = 0.876), despite the larger number of males with suspected allergic symptoms referred to our laboratory each year between 2008 and 2011 (overall male to female ratio = 1.79) (Table 2). By individual allergens, however, the male subjects showed higher positive rates of sIgE test to *Der farinae* (χ2 = 6.82, p < 0.05), cow’s milk (χ2 = 17.51, p < 0.05), crab (χ2 = 5.16, p < 0.05) and shrimp (χ2 = 10.37, p < 0.05), compared with the females (Figure 1). There was no difference in positive sIgE test results between both genders in relation to the other 6 aeroallergens and 5 food allergens (all p >0.05).

**Table 2:** **Number of** p**atients referred for sIgE measurement between 2008 and 2011 [n (%)]**

	**2008**	**2009**	**2010**	**2011**	**Total**
Males (%)	529 (64.99)	976 (62.72)	1447 (64.46)	1571 (64.46)	4523 (64.18)
Females (%)	285 (35.01)	580 (37.28)	793 (35.40)	866 (35.54)	2524 (35.82)
M/F ratio	1.86	1.68	1.82	1.81	1.79

### Age-specific sIgE reactivities to aeroallergens and food allergens in the study population

**Figure 2:**
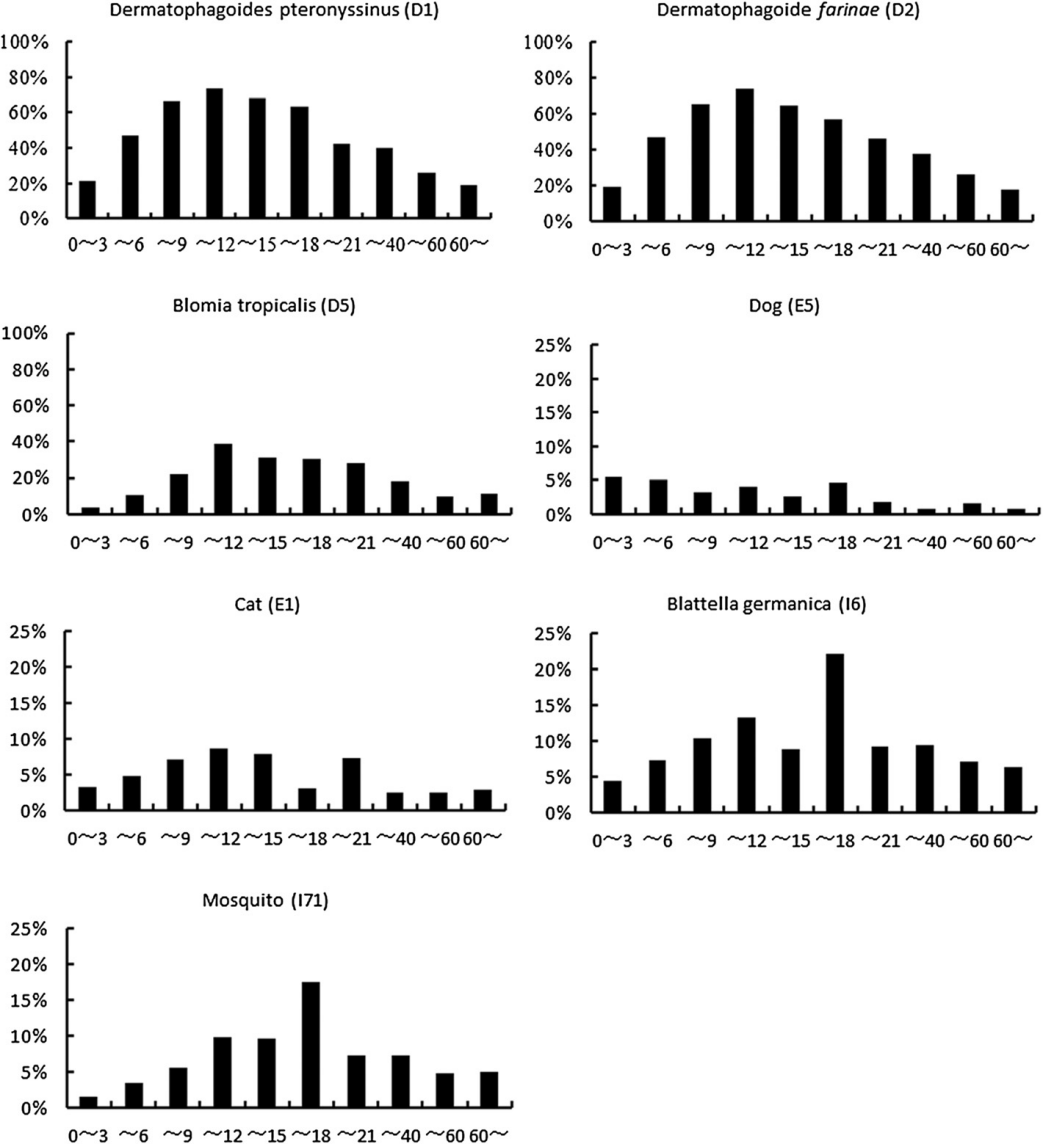
**Positive rates of sIgE to aeroallergens in all age groups.** The total number of cases in each age group: age 0–3 (n = 2695), 4–6 (n = 1887), 7–9 (n = 543), 10–12 (n = 314), 13–15 (n = 113), 16–18 (n = 63), 19–21 (n = 54), 22–40 (n = 683), 41–60 (n = 555), > 60 (n = 140).

Among the 7,047 patients, the prevalence of sensitization to *Der pteronyssinus* (D1), Der farinae (D2) and *Blomia tropicalis* (D5) peaked in the age group older than 9 and below 12 years old. Specifically, both D1 and D2 caused a peak of sensitization in children aged 11 (75.3%, 73 out of 97 cases), compared with the peak of D5 found in children aged 10 (34.9%, 51 out of 146 cases). The positive rate of sIgE to D1 or D2 was higher than that to D5 in any given age group. The prevalence of sensitization to *Blattella germanica* was similar to the findings for mosquito, both with a peak value at the age group older than 15 and below 18 years. The positive rates of the tests for cat dander (E1) and dog hair (E5) were comparable and largely low in all age groups, with several higher rates noted in age groups below 21 (Figure 2).

**Figure 3:**
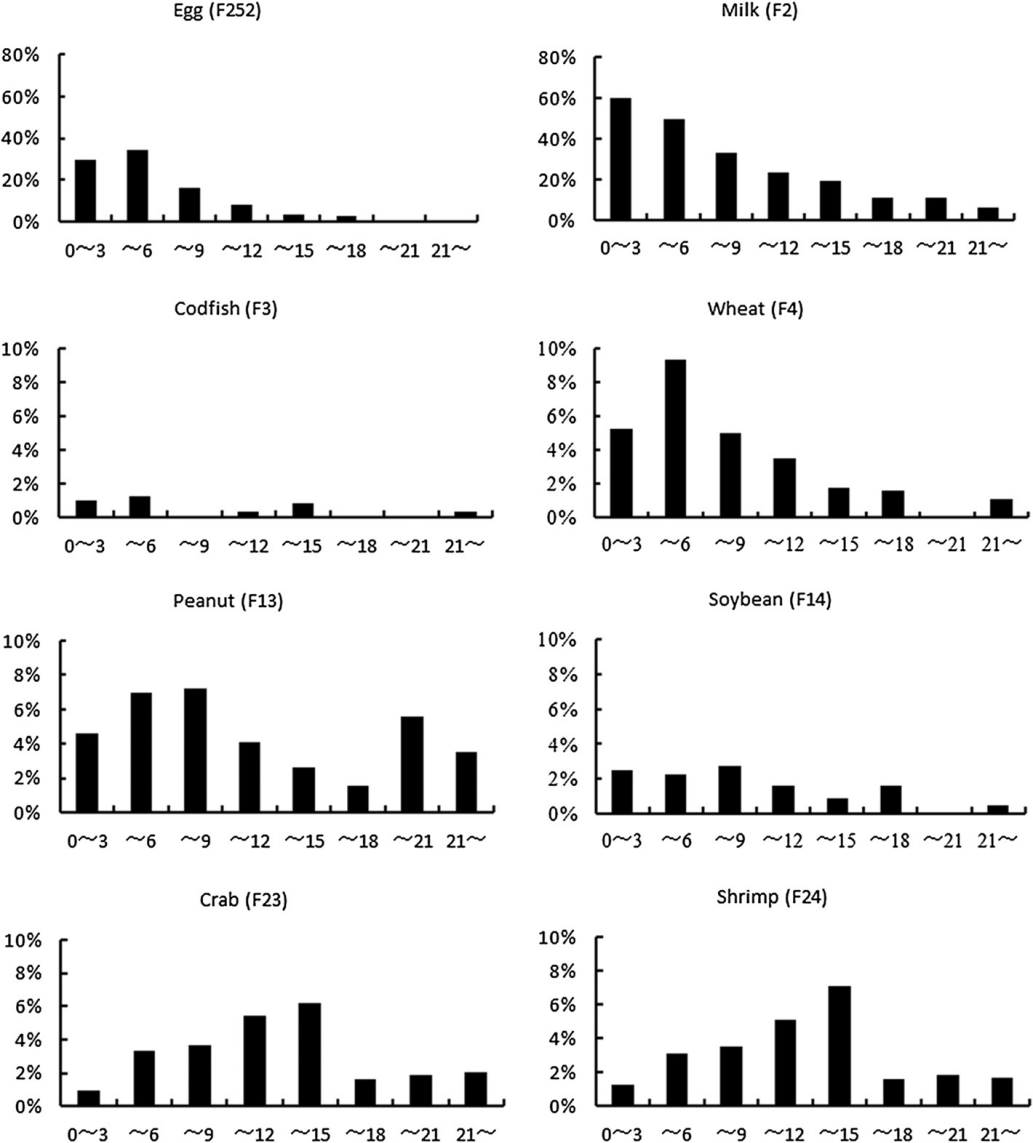
**Positive rates of sIgE to food allergens in all age groups.** The total number of cases in each age group: age 0–3 (n = 2695), 4–6 (n = 1887), 7–9 (n = 543), 10–12 (n = 314), 13–15 (n = 113), 16–18 (n = 63), 19–21 (n = 54), and > 21 (n = 1378).

The positive rates of sIgE to food allergens in all age groups are shown in Figure 3. Apparently, positive response to food allergens was more likely to occur during childhood and teenagers below 18 years, and was fairly infrequent in older age groups. There was a peak of sensitization to cow’s milk among young children aged 3 years or below, and another to eggs or wheat flour at the age group above 3 and below 6 years, followed by increasing tolerance to these food allergens along with older age. Specifically, we found that children at 3 years of age had the highest prevalence of egg sensitization [42.2% (281/666)], and those at 2 years of age were more likely to be allergic to cow’s milk [67.1% (478/712)] compared with other age groups. The positive rates of sIgE to wheat, codfish, peanut, soybean, crab and shrimp were below 10% in each age group. The prevalence of sensitization to crabs and shrimps was comparable between each other in all age groups, both peaking in the age group above 12 and below 15.

**Figure 4:**
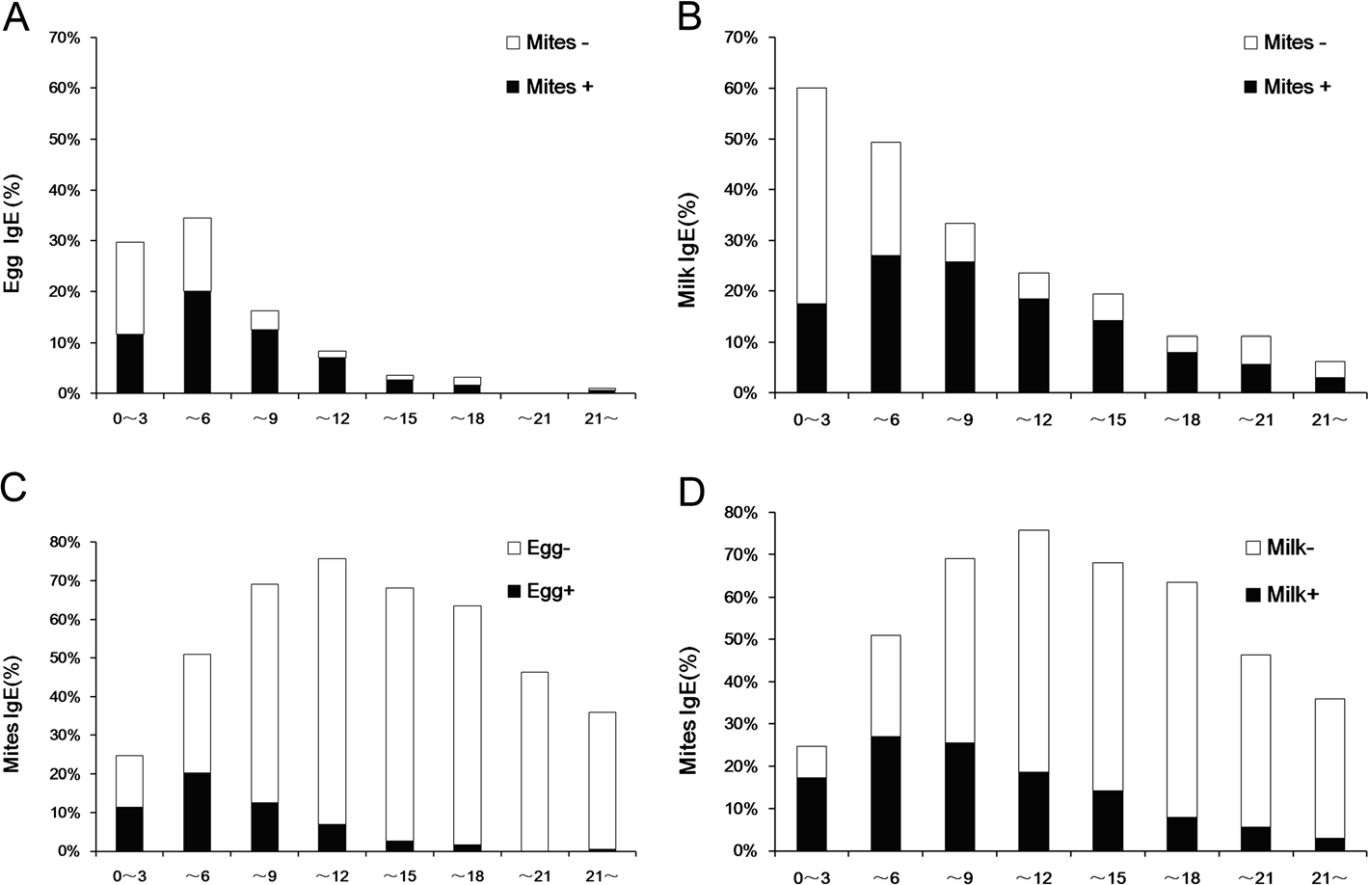
**Correlation between sensitization to food allergens (eggs and cow’s milk) and to house dust mites (Der pteronyssinus, Der farinae, and Blomia tropicalis) along with age.** Panels **A** and **B**: Percentage of positive sIgE response to any house dust mite (*Der pteronyssinus, Der farinae,* and *Blomia tropicalis*) among egg sIgE-positive (Figure 4A) or cow’s milk sIgE-positive (Figure 4B) subjects in each age group. Panels **C** and **D**: Percentage of positive sIgE response to eggs (Figure 4C) or cow’s milk (Figure 4D) among subjects with positive sIgE response to any house dust mite (*Der pteronyssinus, Der farinae,* and *Blomia tropicalis*) in each age group. Black bars: sIgE-positive; Hollow bars: sIgE-negative.

Along with older age in the present study, there was an ascending tendency in the overall positive rate of sIgE response to any of house dust mites (*Der pteronyssinus*, *Der farinae*, and *Blomiatropicalis*) in subjects who tested positive for sIgE to eggs (Figure 4A) or cow’s milk (Figure 4B). Interestingly, the percentage of positive house dust mite sIgE response among egg or cow’s milk sIgE-positive subjects steadily increased along age groups and peaked in the age group above 9 and below 12 years (84.62% and 78.38%, respectively) (Figures 4A, B), in consistency with the peaks of sensitization to *Der pteronyssinus*, *Der farinae*, and *Blomiatropicalis* as found in our study. Conversely, there was a descending tendency in the positive rate of sIgE response to eggs (Figure 4C) or cow’s milk (Figure 4D) in subjects who tested positive for sIgE to any house mite (*Der pteronyssinus*, *Der farinae*, and *Blomiatropicalis*) along with older age. In age groups older than 18, there were actually very few egg sIgE-positive (8/1432, 0.56%) or cow’s milk sIgE-positive (44/1432, 3.07%) cases among those who tested positive for sIgE to any house dust mite (Figures 4C, D).

### Correlation analysis of positive allergen-specific sIgE in the study population

Five aeroallergens with class 3 or higher sIgE responses (D5, I6, E1, E5 and I71), D5 in particular, correlated well with positive sIgE responses to two common species of house dust mites (*Der pteronyssinus* and *Der farinae*) (Table 3). Except for dog hair (E5), more than 73% of subjects with ≥ class 3 sIgE responses to remaining four aeroallergens (D5, I6, E1, and I71) tested positive for concomitant sIgE response to *Der pteronyssinus* (D1) and *Der farinae* (D2). Moreover, good correlation in positive sIgE response was found between D1 and D2 (r = 0.919) (p < 0.001).

**Table 3:** Correlation of positive sIgE responses (class 3 or higher) among aeroallergens

**Allergens**	**Cases with class 3 or higher sIgE response**	**Cases concomitantly with positive sIgE response to D1 or D2**	
		**D1 (%)**	**D2 (%)**
D5	112	111 (99.1)	111 (99.1)
I6	28	23 (82.1)	21 (75.0)
E5	7	1 (14.3)	1 (14.3)
E1	33	26 (78.8)	28 (84.8)
I71	26	20 (76.9)	19 (73.1)
D1	1654	—	1647 (99.6)
D2	1701	1685 (99.1)	—

By kappa test, the strongest agreement in positive sIgE tests was between D1 and D2 (kappa = 0.838, p < 0.001), followed by F23 and F24 (kappa = 0.653, p < 0.001). There was also a significant agreement in positive sIgE test between I6 and I71 (kappa = 0.572, p < 0.001), but not between E1 and E5, F252 and F2, or F13 and F14 (all p > 0.05).

## Discussion

Sensitization to allergens has been recognized as the most important risk factor for allergic diseases which are increasing in prevalence worldwide each year [3]. As epidemiological surveys have shown, dust mites are the most important allergens in China [4], compared to pollens, dust mites and animal furs in European and American countries [5,6]. While human allergies can be attributed to thousands of allergens, the distribution of these allergens may vary in relation with different geographical regions and age groups. By using the REAST protocol for laboratory measurement of a combination of serum specific IgEs which were most commonly reported in previous sporadic local surveys, the present study was the first attempt to have a close look at the distribution of allergens during the recent years in region of Guangzhou, the largest city in southern China, from which derives the large group of subjects (n = 7,047) with suspected allergic symptoms included in this study.

Among the 15 common allergens in our study, *Der pteronyssinus* and *Der farinae* were demonstrated to be the most common aeroallergens, while eggs and cow’s milk to be the most common food allergens, responsible for higher overall positive rates of sIgE response (>20%) in the study population. By classification of sIgE reactivities, a large percentage of the positive sIgE tests (>70%) to any of the remaining allergens showed low-class (class 1 or 2) responses. Based on laboratory criteria, these low-class responses corresponding to a sIgE level between 0.35 and 3.5 IU/mL may be labelled as weak or even suspected positive results, and are therefore inadequate to determine the diagnosis of allergy. However, all subjects in the present study had been evaluated by physicians to have high suspect of allergies before referring to our laboratory for sIgE measurement. Given the conspicuous symptoms in these subjects, we speculated that the presence of allergic disease should not be neglected simply because of low-class laboratory response, and it should be considered with reference to clinical findings.

With respect to gender, although the male subjects were more likely to be allergic to *Der farinae*, cow’s milk, crab and shrimps than the females, we failed to found a significant difference in the overall rate of positive sIgE tests between male and female subjects in the present study. However, we understood that, by no means this could be directly translated into the comparable sensitization to allergens between both genders in the real world. During the present study, there were obviously more male than female subjects with suspected allergic symptoms referred to our laboratory each year (male to female ratio: 1.68 to 1.86). Given that nearly 80% of the subjects in our study aged below 15, the larger number of referred males may partly suggest that childhood allergies are more prevalent in boys than in girls.

The present study did not detect serum total IgE. In a Korean study, total IgE levels were shown to be higher in males. Particularly, total IgE levels in girls increased with age from 3 to 6 years, while a plateau was reached in boys during the same age range. These findings suggested a disparity in total IgE development during early years of life between boys and girls [7]. The significant difference in the number of clinical referrals between genders in our study may raise an interest for further investigations on epidemiology and underlying mechanisms.

Among 30 or so species of mites linked to human allergies, *Der pteronyssinus*, *Der farinae*, *Blomia tropicalis*, and *Euroglyphusmaynei* have been reported to be more closely associated with these conditions [8,9]. In fact, house dust mites grow well in any places with sufficient humidity, warmth and food sources. In addition to indoor environments, mites or mite allergens have been detected in many outdoor locations in southern China [10]. In Guangzhou, situated in the subtropical zone, previous studies have identified *Der pteronyssinus* and *Der farinae* as the prevailing source of mite allergens in house dusts. In our tests for aeroallergens, higher positive rates were found for *Der pteronyssinus* and *Der farinae*, followed by *Blomiatropicalis*, with the highest overall positive rate linked to *Der farinae* (50.72%), and the highest rate of strong positive sIgE response (class 5 or 6) linked to *Der pteronyssinus* (19.06%). The results of the present study also showed that the prevalence of sensitization to *Der farinae*, *Der pteronyssinus* or *Blomiatropicalis* was highest between the ages of 9 and 12, with the peaks for *Der farinae* and *Der pteronyssinus* noted in children aged 11, and for *Blomiatropicalis* in children aged 10. This observation was similar to data from a large-sample Japanese study on sIgE reactivities to common allergens, which demonstrated high positive rates to indoor house dusts and mites in the study population and a peak of sensitization in children aged 10 [11]. Compared with northern regions, the humid, warm environment in southern China plus the common use of enclosed indoor air-conditioning for a longer period in a year may favour the optimal growth of mites and regularly expose to mite allergens.

In a study on skin prick tests given to 85 asthmatic children in Atlanta, USA, Melody and colleagues identified allergy to cockroach in 48% of the subjects, and concluded that cockroach was also a major indoor allergen immediately following dust mites [12]. While this was true according to the ranking of positive rates in the present study, only 7.12% of our subjects tested positive to *Blattella germanica*. The reason for such a low prevalence of sensitization may be that nearly 80% of the subjects aged below 15, whereas the peak for sensitization to German cockroach was identified in the age group above 15 and below 18. Nevertheless, there was a good correlation between sensitization to several aeroallergens that caused ≥ class 3 sIgE responses (such as *Blattella germanica* and mosquito) and sensitization to the two major allergens (*Der pteronyssinus and Der farinae*). Based on these findings, we speculated the probable presence of cross reactions among German cockroach, mosquito and house dust mites. This echoed one of our previous studies [13] which suggested significant cross reactivity between allergens of cockroach and dust mite, as reflected by the concomitant sensitization to dust mites among individuals allergic to cockroach, and the co-existence of sensitization to *Blattella germanica* and dust mites among a number of asthmatic patients.

Clinical data have demonstrated that about 80% of asthmatic children are sensitized to one or more aeroallergens [14]. In addition to pollens, dust mites, moulds and cockroach, animal allergens such as cats and dogs in close contact with human living environment are important offending factors responsible for pathogenesis of allergic diseases. It was estimated that nearly 49% of families in the United States keep dogs or cats as pets, and about 25% in the United Kingdom and Germany raise cats [15]. With the booming economy and improving living standards in China, pet-raising has been increasingly popular in Chinese families. In this study we found much higher sIgEreactivities to cat dander than to dog hair among the 7,045 serum samples, as indicated by 33 cases of ≥ class 3 sIgE responses to cat dander versus only 7 to dog hair. Moreover, sensitization to cat dander was more closely correlated with sensitization to *Der pteronyssinus and Der farinae*, compared with dog hair (Table 3).

Of the 8 common food allergens in this study, cow’s milk and eggs were associated with first two highest, and fish with the lowest rate of positive sIgE responses. The immature digestive and immune functions in newborns and young children, hence the vulnerability of their guts to food allergens, may explain the high positive rate of sIgE response to cow’s milk which is usually among the earliest foods given to babies. The present study showed that there was a peak of sensitization to cow’s milk among young children aged 3 years or below, and another peak to eggs at the age group above 3 and below 6 years, followed thereafter by increasing tolerance to these food allergens along with older age. These findings were in line with a large-sample study on positive rates of sIgE antibody to allergens in Japan [16]. Furthermore, we found that children at 3 years of age had the highest prevalence of egg sensitization, and those at 2 years of age were more likely to be allergic to cow’s milk compared with other age groups. This suggested that, since sensitization to eggs and cow’s milk occurs mostly during early years of life, infants and young children should be monitored closely for food allergy in order to facilitate prevention of this condition. In the present study, the positive rates of certain allergens, such as wheat flour, peanut, soybean, codfish, crab and shrimp, largely corresponded with a suspected or mild allergic response. Data on allergy to wheat were limited but showed similarities with allergy to barley. Unlike cow’s milk and egg, allergy to peanut usually lasts a lifetime [17], and only 10% of the sensitized children will develop tolerance to peanut as they grow up [18]. Our study also showed that comparable prevalence of sensitization to crabs and shrimps in all age groups, with the highest prevalence being in the age group above 12 and below 15. This may be explained by the potential cross-reactivity between crab and shrimp [19] resulting from presence of common epitopes among many allergens [20]. Studies have shown that occurrence of allergic symptoms correlated with level of serum specific IgE antibody and exposure to allergens. In particular, the prevalence of asthma was considerably high among subjects with high levels of serum sIgE [21]. This suggests that measurement of serum sIgE can be helpful for diagnosis of allergic disease in clinical practices.

While skin prick tests (SPTs) represent the first level of allergy diagnosis, reliability of SPT depends substantially on the skillful manipulation of allergists (in Guangzhou, licensed specialist nurses), recent use of histamines and good compliance of the tested subject (which is very difficult to achieve in young children). When these are not fully ensured or standardized as the subjects were referred from many hospitals, the results of SPT would not be adequate to account for an observational study on prevalence of allergen sensitization in a relatively large sample. In contrast, measurement of serum sIgE can provide standardized, safe, and quantitative results. Positive findings of specific IgE plus clinical symptoms can be valuable in confirming a diagnosis of allergy.

The sIgE detection method we used was ALLERG-O-LIQ. According to the studies by various authors, the consistency of ALLERG-O-LIQ with ImmunoCAP results (the most common method used in allergy diagnosis) for quantitative or semi-quantitative determination of various allergens has been verified [22-24], although the correlation is stronger for aeroallergens than for food allergens.

## Conclusions

In summary, the present study showed that *Der pteronyssinus*, *Der farinae*, cow’s milk and eggs are the major allergens responsible for allergic diseases in Guangzhou, southern China. In our study population, allergies were mostly caused by cow’s milk and eggs early in life, and then gave place to the increasing prevalence of sensitization to *Der pteronyssinus* and *Der farinae* at older age. Some children may grow out of cow’s milk or egg sensitization, or experience a switch in the offending allergens from cow’s milk and eggs to aeroallergens (especially the house dust mites) as they get older. Such a sequence of events may be a result of allergy march [25]. Knowledge on the prevalence of allergen sensitization in different age groups would help early diagnosis and intervention of allergic diseases in this large geographical region.

## Availability of supporting data

The data set supporting the results of this article is included within the article.

## Competing interests

The authors have no conflicts of interest related to this article.

## Authors’ contributions

BQS and GQZ designed and supervised the study; PYZ and HMH performed all the measurement; PYZ and GQZ completed the manuscript; DHC and XWZ reviewed the study and participated in data analysis. All authors read and approved the final manuscript.
